# From the perspective of prolactin: a view on obesity

**DOI:** 10.3389/fendo.2026.1762596

**Published:** 2026-03-16

**Authors:** Yun Wang, Dan Luo, Guowei Fang, Muzi Ge, Yanqin Huang, Hualiang Deng

**Affiliations:** 1 The First Clinical Medical College, Shandong University of Traditional Chinese Medicine, Jinan, Shandong, China; 2 Department of Endocrinology, Affiliated Hospital of Shandong University of Traditional Chinese Medicine, Jinan, Shandong, China; 3 Department of Gastroenterology, Affiliated Hospital of Shandong University of Traditional Chinese Medicine, Jinan, Shandong, China

**Keywords:** BMI, dopamine agonists, hyperprolactinemia, metabolic syndrome, obesity, prolactin, weight loss

## Abstract

The physiological roles of prolactin extend beyond its classical functions in reproductive regulation. Emerging evidence indicates that prolactin is involved in energy homeostasis and may interact pathophysiologically with obesity; this has attracted increasing attention in endocrinology and metabolic research. Hyperprolactinemia (HPRL) is frequently observed in obese individuals. Observational studies have reported that weight-loss interventions are associated with reduced circulating prolactin levels, whereas dopamine agonists, which suppress prolactin secretion, improve metabolic and endocrine abnormalities in patients with established hyperprolactinemia. Accumulating evidence suggests an association between hyperprolactinemia and obesity. However, the directionality and causality of this relationship remain unclear. Experimental and translational studies suggest that elevated prolactin levels contribute to obesity-related phenotypes through multiple pathways, including altered central appetite regulation, modulation of adipocyte differentiation and lipid storage, impairment of insulin sensitivity, and disruption of the hypothalamic–pituitary–gonadal (HPG) axis. In contrast, obesity may be associated with increased circulating prolactin levels, which are potentially mediated by adipose tissue expansion, enhanced aromatase-dependent estrogen production, and chronic low-grade systemic inflammation. This review aimed to provide a systematic synthesis of current evidence regarding the mechanistic links between hyperprolactinemia and obesity, with an emphasis on the biological properties of prolactin, clinical characteristics of obesity complicated by HPRL, and molecular and physiological pathways underlying their reciprocal interactions. In addition, we critically evaluate current clinical management strategies, including dopamine agonist therapy and lifestyle-based weight-loss interventions, highlighting existing uncertainties and future directions aimed at improving the diagnosis and integrated management of these frequently coexisting conditions.

## 1 Introduction

Moderate elevations in circulating prolactin have been associated with favorable metabolic effects (1), whereas both hyperprolactinemia and hypoprolactinemia have been linked to adverse metabolic outcomes (2). Clinically, hyperprolactinemia is defined as a serum prolactin concentration exceeding 25 ng/mL and is classified as either physiological (transient) or pathological. Pathological hyperprolactinemia most commonly results from prolactin-secreting pituitary adenomas (prolactinomas) but may also arise from ectopic prolactin production, chronic kidney disease, or certain medications (3).

The global prevalence of overweight and obesity continues to rise and represents a major public health concern (4). Obesity is a chronic, progressive disorder characterized by systemic dysregulation of energy homeostasis and is associated with numerous adverse health outcomes (5). In addition to excess adiposity, obesity involves interconnected pathological processes, including dyslipidemia, insulin resistance, chronic low-grade inflammation, and adipose tissue hypoxia. Chronic low-grade inflammation is a hallmark of obesity-related adipose tissue expansion and contributes substantially to peripheral metabolic dysfunction (6). Obesity is a well-established risk factor for multiple comorbidities, including metabolic syndrome, type 2 diabetes mellitus, and several obesity-related malignancies (7).

Several studies have demonstrated a positive association between serum prolactin levels and body mass index (BMI), suggesting a potential role for hyperprolactinemia in obesity pathophysiology (8). Dopamine agonists, such as bromocriptine and cabergoline, are first-line therapies for hyperprolactinemia and effectively normalize prolactin levels (9). Clinical studies have also reported improvements in glycemic control, insulin sensitivity, and lipid profiles in patients treated with dopamine agonists. However, it remains unclear whether these metabolic benefits result directly from prolactin reduction or from broader dopaminergic and neuroendocrine effects. This review aims to systematically examine the bidirectional relationship between prolactin dysregulation and obesity, integrating mechanistic, clinical, and therapeutic evidence to clarify its pathophysiological and clinical significance.

## 2 Prolactin: biological characteristics

### 2.1 Structure and secretion regulation

Human mature prolactin is a 199-amino-acid polypeptide with an estimated molecular mass of ~23 kDa (10). In healthy individuals, prolactin-secreting cells, predominantly lactotrophs, constitute approximately 15–25% of the total anterior pituitary cell population. This proportion exhibits minimal sexual dimorphism and remains relatively stable across age groups (11). The anterior pituitary is the primary site of prolactin synthesis and secretion. Moreover, prolactin can be synthesized locally in the extrapituitary tissues via autocrine and paracrine mechanisms. Prolactin is expressed in multiple peripheral tissues including the mammary glands, ovaries, prostate, adipose tissue, and immune cells (12). Although the anterior pituitary is the principal source of circulating prolactin, early studies reported persistently low but detectable serum prolactin levels following hypophysectomy, providing indirect evidence of physiologically relevant extrapituitary prolactin production (13).

Prolactin-secreting cells (lactotrophs) in the anterior pituitary gland are the primary sources of prolactin synthesis and secretion. Furthermore, gene transcription is regulated by promoter and repressor elements. At the molecular level, the prolactin gene contains two distinct promoter regions: the proximal promoter drives basal and regulates prolactin transcription, predominantly in pituitary lactotrophs, whereas the distal promoter is implicated in tissue-specific extrapituitary prolactin expression (14). Prolactin secretion is under stringent neuroendocrine control, with tonic inhibition mediated by tuberin fundibular dopaminergic (TIDA) neurons constituting the principal regulatory mechanism. Elevated circulating prolactin levels activate TIDA neurons via a short-loop negative feedback circuit, resulting in increased dopamine (DA) release into the hypophyseal portal vasculature. Dopamine then binds to D_2_ receptors on lactotrophs, leading to the suppression of prolactin gene transcription and hormone secretion (15).

In addition to dopaminergic inhibition, prolactin secretion is modulated by a diverse array of hypothalamic factors including vasoactive intestinal peptides, serotonin, histamine, oxytocin, estrogen, thyrotropin-releasing hormone, dopamine receptor antagonists, and epidermal growth factor (16). Furthermore, autocrine/paracrine signals from the pituitary gland as well as humoral and neural inputs from peripheral organs can exert either inhibitory or stimulatory effects on prolactin release (17). Temporal regulation of prolactin secretion is also coordinated by the suprachiasmatic nucleus, which integrates circadian and photoperiodic information to fine-tune the timing of prolactin surges, particularly during physiologically relevant states such as mating, lactation, and seasonal reproductive cycles (18). Collectively, the interplay between endogenous and exogenous regulators of prolactin concentration is highly complex with overlapping, context-dependent, and often non-linear causal relationships, posing significant challenges for precise quantification and interpretation. Consequently, in the clinical evaluation of patients with hyperprolactinemia, careful consideration of potential confounding factors, including medication use, stress, sleep disruption, renal or hepatic dysfunction, and assay-related variability, is essential to enhance diagnostic accuracy and inform evidence-based therapeutic decision-making.

### 2.2 Target site

The biological effects of prolactin are mediated by the prolactin receptor (PRLR) and its canonical downstream JAK2/STAT5 signaling pathway. Non-canonical pathways (including PI3K/AKT and Ras/MAPK signaling) also contribute to the cellular actions of prolactin (19). Upon ligand-induced activation, phosphorylated PRLR serves as a docking platform for adaptor proteins that recruit components of the PI3K–AKT and Ras/MAPK cascades, thereby modulating immune regulation and inflammatory responses (20). Structurally, PRLR belongs to the type I cytokine receptor family and is expressed as three major splice isoforms—long, short, and intermediate—each exhibiting distinct functional properties, particularly in adipose tissue (21).

The binding of prolactin to the long form of PRLR triggers a cascade of tyrosine kinase-dependent signaling events, including JAK2 autophosphorylation and subsequent phosphorylation of specific tyrosine residues on the receptor cytoplasmic domain (22). JAK2 activation is one of the earliest and most critical steps in long-form PRLR signaling and is essential to propagate downstream signals via phosphorylation of signal transducers and activators of transcription (STATs). STAT1, STAT3, STAT5A, and STAT5B are key STAT family members implicated in PRL signaling. Although the physiological roles of prolactin-induced STAT1 and STAT3 activation remain unclear, STAT5A and STAT5B are central mediators of the diverse biological functions of PRL.

STAT5A and STAT5B are highly conserved homologous transcription factors with amino acid sequences that contain six structurally and functionally distinct domains: an N-terminal domain, coiled-coil domain, DNA-binding domain, linker domain, SH2 domain, and C-terminal transactivation domain. The SH2 domain mediates tyrosine phosphorylation, primarily by JAK2, and subsequent homo- or heterodimerization of STAT5 proteins. The phosphorylation of a conserved tyrosine residue (Tyr694 in STAT5A and Tyr699 in STAT5B) within the C-terminal transactivation domain is a pivotal post-translational modification required for STAT5 activation. Through the integrated function of these domains, STAT5 serves as a critical node in cytokine-induced intracellular signal transduction and nuclear transcriptional regulation (23). Upon phosphorylation, STAT5 dimers rapidly translocate to the nucleus, where they bind to γ-interferon activation sequence (GAS) elements—consensus DNA motifs (TTN5AA)—in the promoter regions of target genes. This drives transcription of prolactin-responsive genes.

Collectively, activation of the long-form prolactin receptor (PRLR) initiates the canonical JAK2/STAT5 signaling cascade, which is the principal pathway underlying the diverse biological actions of PRLR (24). This pathway has been increasingly implicated in the regulation of adipocyte differentiation, lipid metabolism, and systemic energy homeostasis. Furthermore, histological and molecular studies have confirmed PRLR expression in human pancreatic β-cells and adipocytes, providing direct anatomical and cellular evidence supporting prolactin’s role in peripheral metabolic regulation (9). Moreover, murine models of PRLR deficiency exhibit impaired adipose tissue development, diminished white adipose tissue mass, and reduced abdominal fat accumulation, collectively suggesting that PRLR signaling critically contributes to the maintenance of adipose tissue homeostasis (25).

### 2.3 Metabolic effects of physiological hyperprolactinemia in pregnancy and lactation

Physiological elevations in prolactin levels, including pituitary-derived prolactin and placental lactogen, preferentially induce hypothalamic leptin resistance in females, thereby promoting increased caloric intake and gestational weight gain. Notably, analogous effects have not been consistently demonstrated in males, underscoring the sex-specific nature of neuroendocrine adaptations (26). This female-predominant response aligns with the marked peripartum surge in prolactin and is integrated within a broader suite of coordinated metabolic adjustments: hypothalamic leptin resistance, heightened appetite, augmented adipose tissue deposition, compensatory expansion of pancreatic β-cell mass, and transient reduction in insulin sensitivity. These adaptations serve to optimize maternal energy storage and ensure a robust nutrient supply to meet the heightened metabolic demands of fetal growth and postpartum milk production (27).

Lactation represents a period of exceptionally high metabolic demand during the mammalian life cycle. During this phase, prolactin acts synergistically with other endocrine factors and local regulatory mechanisms in mammary glands to orchestrate systemic and tissue-specific metabolic adaptations (28). Specifically, prolactin promotes the preferential partitioning of key metabolic substrates, including glucose, amino acids, and lipids, for milk synthesis. Furthermore, prolactin enhances lipid mobilization and transport from adipose tissue to the mammary gland, while simultaneously suppressing *de novo* lipogenesis and lipid uptake in adipocytes and augmenting the lipid biosynthetic capacity in mammary epithelial cells (29). Although overt systemic insulin resistance is generally absent during lactation, adipose tissue exhibits well-documented, selective insulin resistance, a physiological adaptation thought to facilitate lipid mobilization and prioritize nutrient allocation to the mammary glands (30). Although the metabolic actions of prolactin during pregnancy and lactation are sometimes characterized as “diabetogenic,” these effects are more accurately understood as adaptive and evolutionarily conserved responses tailored to meet the heightened metabolic demands of the maternal–offspring unit. In particular, prolactin-mediated regulation of pancreatic β-cell proliferation during pregnancy plays a critical role in expanding functional β-cell mass, thereby supporting maternal glucose homeostasis. This interpretation is substantiated by preclinical studies: mice with heterozygous prolactin receptor deficiency or β-cell-specific deletion of the prolactin receptor exhibit impaired β-cell expansion and increased susceptibility to gestational diabetes mellitus. Collectively, these findings underscore the essential contribution of prolactin signaling to metabolic adaptation throughout pregnancy (31).

## 3 Clinical challenges

Clinical studies have consistently reported the association between hyperprolactinemia and obesity. Furthermore, observational studies have indicated that individuals with elevated prolactin levels tend to exhibit higher adiposity and metabolic dysregulation. For example, obese women demonstrate significantly higher total 24-hour prolactin secretion than normal-weight controls (32). In addition, serum prolactin concentrations significantly positively correlate with both subcutaneous fat area and visceral fat area (33).

Comparative analyses have further revealed that patients with hyperprolactinemia have a higher BMI, fasting plasma glucose, fasting insulin levels, and indices of insulin resistance than normoprolactinemic controls. Correlation analyses across these studies have consistently demonstrated positive associations between circulating prolactin concentrations and BMI, fasting glucose levels, insulin levels, and measures of insulin resistance. Based on these findings, Al Sabieh et al. proposed that weight gain may represent an independent clinical feature of hyperprolactinemia, and reported a markedly increased prevalence of obesity in affected patients (34).

Consistent with these observations, several case series and retrospective reports have identified unexplained early weight gain, and in some cases, presentation of symptoms in patients later diagnosed with prolactinomas (35). In a cohort study of 35 women with prolactinomas, Nunes et al. observed a significant association between hyperprolactinemia and weight gain in 19 patients (36), corroborating earlier findings reported by Greenman et al. (37).

In a large retrospective analysis of 219 patients newly diagnosed with pathological hyperprolactinemia, Colao et al. reported that weight gain was the most frequently observed symptom. The prevalence of weight gain was 94% in women with macroprolactinomas, 59% in those with microprolactinomas, and 53% in patients with non-neoplastic hyperprolactinemia (e.g., functional or drug-induced hyperprolactinemia). Among men, the corresponding prevalence rates of macroprolactinomas and microprolactinomas were 53% and 19%, respectively (38).

## 4 Mechanisms by which hyperprolactinemia may promote obesity

**Figure 1:**
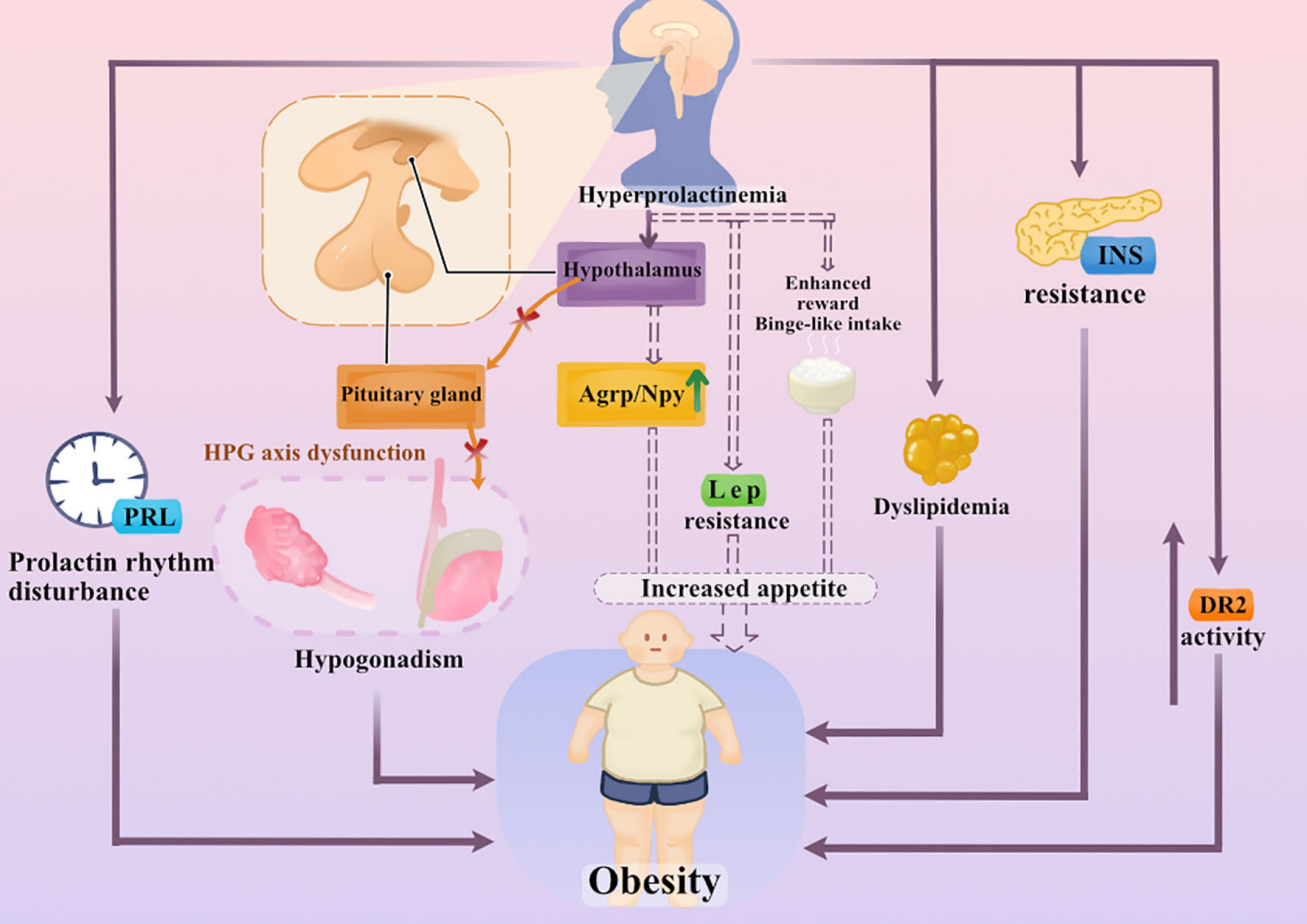
Proposed and experimentally supported mechanisms linking hyperprolactinemia and obesity. Dashed lines indicate increased appetite, leptin resistance, and binge-like intake, mainly derived from rodent studies, and are speculative associations. Arrows represent proposed or experimentally supported mechanisms; causal relationships in humans remain unproven. Bidirectional arrows reflect potential reciprocal interactions.

Hyperprolactinemia has been repeatedly cited as being associated with weight gain in both clinical observations and experimental studies; however, the underlying pathogenic mechanisms are extraordinarily complex and multifactorial. Hyperprolactinemia does not function through a single linear pathway but is more likely to affect weight homeostasis through the synergistic regulation of multiple systems. These systems include central neural circuits involved in appetite regulation, satiety signals, and energy homeostasis, as well as peripheral metabolic processes that regulate adipose tissue function, insulin sensitivity, and gonadal axis activity. Furthermore, alterations in the diurnal rhythm of prolactin secretion and the dysregulation of dopaminergic neurotransmitters may promote obesity-related phenotypes in hyperprolactinemia. We, therefore, aimed to systematically review the molecular and pathophysiological mechanisms of hyperprolactinemia and obesity, focusing on key central and peripheral pathways, supported by current experimental and clinical evidence (**Figure 1**).

### 4.1 Central regulation of appetite and energy homeostasis

Patients with hyperprolactinemia may experience increased appetite, leading to higher energy intake and obesity (20). Animal studies have shown that prolactin can regulate the central neural circuits involved in appetite and energy homeostasis. Georgescu et al. used calcium imaging to show that most neurons expressing prolactin receptors in the arcuate nucleus (ARC) of the mouse hypothalamus rapidly undergo calcium-dependent activation under prolactin stimulation, suggesting that the acute effect mediated by calcium ion signaling is an important central mechanism by which prolactin regulates appetite and energy balance (39). Neuropeptide Y (NPY) and agouti-related protein (Agrp) are key appetite-promoting factors in ARC neurons, and their upregulation is strongly associated with increased food intake. Dopamine is also an important factor that inhibits prolactin secretion. In a study on female mice lacking dopamine D2 receptors (lacDrd2KO) characterized by chronic hyperprolactinemia, Agrp and NPY expression were significantly increased in the ARC, and NPY expression was increased in the dorsomedial nucleus (DMN) (40). However, in rodents, these neuroendocrine effects are associated with enhanced feeding signaling and increased food intake; however, their relevance to human physiology remains to be fully confirmed.

In addition to homeostatic feeding regulation, hyperprolactinemia may affect reward-driven feeding behavior. In rodent binge-eating models, lacDrd2KO mice consumed approximately twice the amount of high-fat food as controls, accompanied by enhanced activation of dopaminergic neurons in the mesolimbic reward circuit (41). These results suggest that chronic hyperprolactinemia may enhance the responsiveness to reward foods in rodents; however, direct evidence for its causal role in humans is lacking.

Furthermore, prolactin may interfere with the central leptin signaling pathway (42). Under physiological conditions, leptin exerts appetite-suppressive effects by inhibiting Agrp and NPY neurons in the ARC (43). In rodent models of hyperprolactinemia, leptin-induced STAT3 phosphorylation in the ARC and DMN was significantly reduced, suggesting impaired central leptin signaling (44). Consistent with this view, rats receiving chronic intraventricular (ICV) prolactin infusions did not show a significant reduction in food intake and body weight after central administration of leptin (4 μg). In saline-treated control animals, leptin injection resulted in a significant reduction in both food intake and body weight (P < 0.05) (26). However, whether prolactin induces clinically significant leptin resistance in humans requires further investigation.

### 4.2 Adipose tissue dysfunction and lipid metabolism

Clinical evidence suggests that elevated prolactin levels are strongly associated with disorders of lipid uptake, synthesis, and catabolism (20). Patients with hyperprolactinemia present with an increased body fat percentage and total fat mass, suggesting obesity-related changes in body composition. Compared to healthy controls, these patients have significantly higher levels of serum total cholesterol, low-density lipoprotein cholesterol (LDL-C), and triglycerides, while high-density lipoprotein cholesterol (HDL-C) levels were lower (9).

At the cellular level, prolactin can directly affect adipogenesis by regulating key transcription factor expression. Experimental studies have shown that prolactin upregulates the expression of CCAAT/enhancer-binding protein β (C/EBPβ) and peroxisome proliferator-activated receptor γ (PPARγ), which are involved in the early and terminal stages of adipocyte differentiation, respectively (45). *In vivo* studies further support this effect; mice lacking prolactin receptors showed a significant reduction in adipose tissue mass and the number of adipocytes (25), whereas activation of the long prolactin receptor promoted lipid storage and induced visceral adipocyte hypertrophy (46). Consistent with this, *in vitro* experiments have shown that prolactin receptor (PRLR) gene expression is significantly upregulated during the differentiation of preadipocytes into mature adipocytes, with an approximately 90-fold increase, supporting the functional role of prolactin signaling in adipocyte development and lipid accumulation (47).

In rodent models, pathological hyperprolactinemia also significantly affects brown adipose tissue (BAT), manifested as the downregulation of thermogenic markers, BAT “whitening,” and impaired adaptive thermogenesis. Simultaneously, prolactin can alter the expression of lipidogenesis genes in subcutaneous white adipose tissue and exacerbate glucose intolerance induced by a high-fat diet (48). These results suggest that excessively elevated prolactin levels may trigger or aggravate high-fat diet-induced obesity by impairing BAT function. However, direct evidence regarding prolactin-mediated abnormalities in human BAT function remains limited, and their clinical significance requires further clarification.

### 4.3 Insulin resistance and glucose metabolic dysregulation

Clinical, epidemiological, and experimental studies have consistently shown that hyperprolactinemia is strongly associated with glucose metabolism disorders that primarily manifest as insulin resistance and impaired glucose tolerance. Insulin resistance constitutes an important mechanistic link between prolactin abnormalities and obesity-related metabolic disorders, and is a core pathological feature of metabolic syndrome and type 2 diabetes (49).

A hyperglycemic clamp test showed that individuals with higher circulating prolactin levels had significantly reduced insulin sensitivity (50). Consistent with this, hyperprolactinemia is associated with impaired glucose tolerance and a reduced insulin response (51). In women, clinical studies have found that women with hyperprolactinemia have significantly lower insulin sensitivity than age- and BMI-matched normal prolactin controls (52); in men, epidemiological data also suggest a positive correlation between serum prolactin levels and insulin resistance (53). Notably, clinical observations have shown that even with routine glucose-lowering therapy, patients with persistent hyperprolactinemia may experience poor glycemic control (54). A longitudinal analysis of the Framingham Heart Study further supports this association: for every 5-μg/L increase in baseline prolactin levels in men, the risk of developing type 2 diabetes increases by approximately 70%, suggesting a significant and sex-differentiated association between prolactin imbalance and long-term impaired glucose homeostasis (55). However, current evidence mainly supports a correlation: whether prolactin affects insulin sensitivity by directly interfering with the insulin signaling pathway or primarily indirectly through fat accumulation, chronic inflammation, and neuroendocrine changes requires further mechanistic investigation.

### 4.4 Hypothalamic–pituitary–gonadal axis disruption

The HPG axis is extremely sensitive to circulating prolactin levels. Hyperprolactinemia interferes with the activity of gonadotropin-releasing hormone (GnRH) neurons and is a recognized cause of functional hypogonadism (56). Elevated prolactin levels can impair the reproductive function through central (neuroendocrine) and peripheral (gonadal) mechanisms.

At the central level, excessive prolactin inhibits the synthesis and pulsatile secretion of hypothalamic gonadotropin-releasing hormone (GnRH), thereby reducing the release of pituitary gonadotropins, luteinizing hormone and follicle-stimulating hormone, ultimately leading to hypogonadism (57). At the peripheral level, hyperprolactinemia can directly inhibit the activity of steroid-related enzymes in ovarian and testicular tissues, impair the synthesis and secretion of sex hormones, and further disrupt overall endocrine homeostasis.

In women, prolactin has a significant inhibitory effect on ovarian steroid production, leading to reduced synthesis and secretion of estradiol and progesterone (58). Estrogen deficiency is strongly associated with unfavorable changes in fat distribution, particularly increased subcutaneous and visceral fat deposition in the abdomen (59). Notably, these metabolic changes are reversible to some extent. Clinical studies have shown that postmenopausal women treated with estrogen alone or estrogen–progestin combination therapy have significantly reduced visceral fat content, decreased fasting blood glucose levels, and improved insulin sensitivity, manifested as decreased insulin levels (60).

In men, hyperprolactinemia is accompanied by hypogonadism and androgen deficiency, which can lead to unfavorable changes in the body composition. Androgen deficiency is characterized by increased fat mass and decreased bone density, which increase the risk of obesity and metabolic dysfunction (61). Expression of prolactin receptors has been detected in Leydig cells, Sertoli cells, and seminiferous epithelium, suggesting that prolactin may regulate testosterone synthesis and spermatogenesis by directly acting on various testicular cell types (62). Therefore, prolactin is increasingly being recognized as an important regulator of testicular function and may play a pathophysiological role in male hypogonadism and infertility. Secondary androgen deficiency can promote adipose tissue accumulation and impair energy metabolism, thereby indirectly contributing to the development and progression of obesity.

### 4.5 Circadian prolactin rhythm and metabolic regulation

Alterations in the prolactin secretion rhythm have been repeatedly demonstrated to be associated with metabolic abnormalities in various physiological and pathological states. As early as the 1980s, clinical studies reported that obese individuals had circadian prolactin secretion rhythm disorders linked to adverse metabolic phenotypes, including insulin resistance, dyslipidemia, and systemic inflammatory responses. Subsequent studies confirmed a significant correlation between prolactin rhythm dysregulation and metabolic dysfunction (63).

Circadian rhythm misalignments, such as those imposed by jet lag, play an important role in the occurrence and progression of metabolic dysfunction-associated fatty liver disease (64). Against this backdrop, phase shifts or amplitude reductions in the prolactin rhythm have been proposed to promote a positive energy balance, weight gain, and ectopic fat deposition, thereby participating in obesity-related pathophysiological processes (63). However, the specific molecular and physiological mechanisms by which prolactin rhythm disorders exert their effects via central and peripheral metabolic regulatory networks remain to be elucidated.

In summary, an intact prolactin diurnal rhythm may play a crucial role in maintaining metabolic homeostasis, whereas its disruption is associated with impaired glucose homeostasis, adipose tissue dysfunction, and an increased risk of metabolic comorbidities. However, rigorously designed experimental and longitudinal clinical studies are still needed to clarify the causal relationship between prolactin diurnal dynamics and overall metabolic health, and to elucidate the underlying mechanisms.

### 4.6 Dopamine signaling impairment in obesity

In obese individuals, diminished dopamine D2 receptor (D2R) activity in the brain may impair the inhibitory control of dopamine on prolactin secretion, thereby contributing to hyperprolactinemia. Under physiological conditions, prolactin secretion from lactotrophs in the anterior pituitary is tonically suppressed via dopaminergic signaling through hypothalamic D2R. Neuroimaging studies have consistently demonstrated reduced D2R availability, reflected by decreased binding potential, in key striatal and extrastriatal regions of obese individuals, supporting an association between obesity, central dopaminergic hypofunction, and dysregulated prolactin homeostasis (32). These findings are consistent with the well-established concept of reduced central dopaminergic tone in obesity. They also parallel the endocrine and metabolic effects of antipsychotic medications, particularly those acting as potent D2R antagonists that are clinically associated with both hyperprolactinemia and weight gain.

## 5 Mechanisms by which obesity may promote hyperprolactinemia

**Figure 2:**
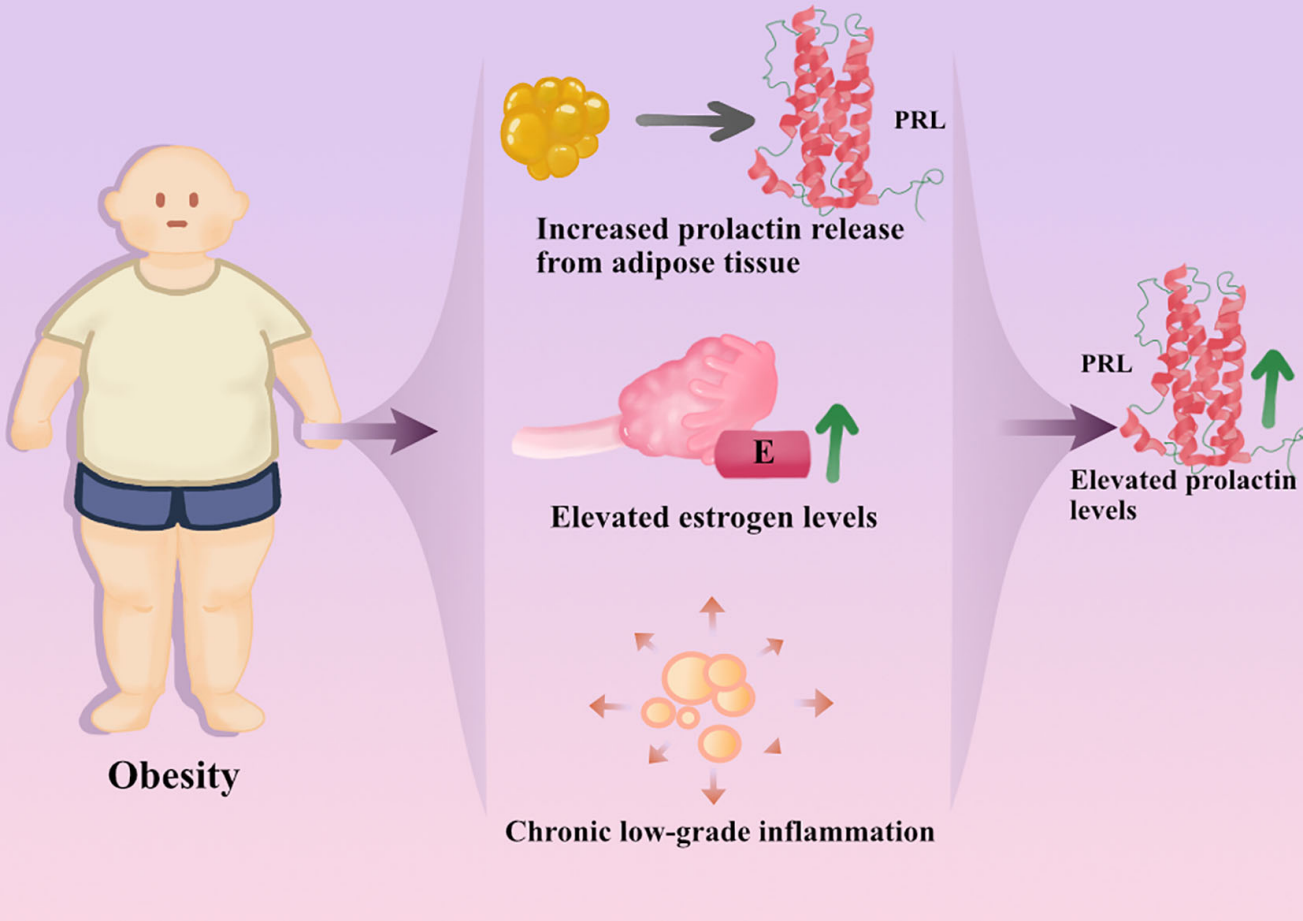
Potential mechanism by which obesity may promote an increase in prolactin secretion. These mechanisms are primarily supported by experimental and observational data.

We have systematically analyzed the potential mechanisms by which hyperprolactinemia promotes obesity. Within this pathological framework, obesity is not merely a downstream consequence of elevated prolactin levels, but may promote increased prolactin secretion. These interactions may collectively form a self-reinforcing vicious cycle, causing hyperprolactinemia and obesity to reinforce and worsen continuously. This bidirectional regulatory process involves multiple interrelated mechanisms, including dysregulation of local prolactin synthesis and secretion (e.g., originating from adipose tissue or other extrapituitary tissues), alterations in prolactin metabolism and clearance processes, and a persistent chronic low-grade inflammatory state (**Figure 2**).

However, the current understanding of these mechanisms is primarily based on experimental studies and relevant clinical observations, and the relative contribution and clinical significance in humans require further investigation. Future studies should focus on in-depth mechanistic investigations and rigorously designed clinical trials to elucidate these mechanisms.

### 5.1 Adipose tissue-related aspects

Previous studies have shown a positive correlation between spontaneous prolactin release and visceral fat mass in obese women. Adipose tissue is an important extrapituitary source of prolactin. Although the absolute amount of prolactin secreted by adipose tissue is significantly lower than that produced by the anterior pituitary gland, the large number of adipocytes (especially in highly obese individuals) suggests that adipose-derived prolactin may significantly contribute to systemic prolactin production (29). Furthermore, prolactin secretion has been consistently demonstrated in various adipose-derived models, including explants from isolated adipose tissue, primary mature adipocytes, and the human LS14 preadipocyte line (derived from subcutaneous and visceral fat deposits). Furthermore, secretion levels gradually increase during prolonged culture. Prolactin production and release have also been demonstrated in human breast-associated adipose tissue, and it has been shown that preadipocytes synthesize prolactin *de novo*, with its expression and secretion strongly stimulated by cyclic adenosine monophosphate (cAMP) enhancers (65).

Therefore, we hypothesized that increased adipose tissue hypertrophy and quantity in obese individuals, leading to increased prolactin release, may ultimately result in hyperprolactinemia. However, to date, little research has been conducted on this topic. Although *in vitro* experiments have confirmed that human adipose tissue secretes prolactin, further experimental and clinical evidence is needed to support this hypothesis.

### 5.2 Aromatase activity and estrogen-mediated prolactin stimulation

Obesity is accompanied by elevated circulating estrogen levels, which may promote prolactin secretion. Adipose tissue is a site of energy storage and an active organ with important endocrine functions. It also expresses aromatase, which converts androgens into estrogen. In obese individuals, the expression and activity of aromatase in the adipose tissue are enhanced, thereby promoting the synthesis of peripheral estrogen and leading to elevated systemic estrogen levels (66).

Prolactin-secreting cells (prolactinocytes) are the main target of 17β-estradiol (E2). E2 regulates gene transcription through the classical genomic signaling pathway by binding to nuclear estrogen receptors. This action upregulates the expression of prolactin mRNA expression, thereby promoting prolactin gene transcription, protein synthesis, intracellular storage, and subsequent secretion, ultimately leading to elevated levels of circulating prolactin levels (67).

### 5.3 Chronic low-grade inflammation and cytokine-mediated prolactin secretion

Sity-related chronic low-grade inflammation may constitute an important mechanistic basis for elevated prolactin levels. Obesity is characterized by adipocyte hypertrophy and proliferation, leading to the expansion of adipose tissue volume (68). In addition to its energy storage function, the adipose tissue is an active endocrine and immune organ that secretes various inflammatory mediators. Hypertrophic adipocytes can promote the infiltration of macrophages into adipose tissue, thereby creating a local microenvironment characterized by a pro-inflammatory state, manifested by increased production of various cytokines, including tumor necrosis factor-α (TNF-α), interleukin-6 (IL-6), and chemokines. Together, these changes maintain a persistent, systemic, and low-grade inflammatory response.

Inflammatory mediators directly regulate prolactin secretion. In rodents, TNF-α and interleukin-1β (IL-1β) both act directly on pituitary prolactinocytes to stimulate prolactin release, a process that differs from the indirect mechanism regulated by hypothalamic dopamine (69). In primary rat pituitary cell culture, TNF-α can induce a rapid increase in intracellular free calcium concentration, and the time course is highly consistent with the increase in prolactin secretion, suggesting that the calcium signaling pathway plays a key downstream effect in the process of inflammatory factors directly acting on prolactin-secreting cells. To further clarify the role of calcium mobilization in TNF-α-induced prolactin release, researchers gave selective pharmacological interventions to regulate TNF-α-triggered calcium kinetics in dispersed cultured pituitary cells. The results showed that calcium channel blockers (such as verapamil) could significantly inhibit basal and TNF-α-induced prolactin secretion; at the same time, pituitary cells cultured under low extracellular calcium conditions also significantly reduced TNF-α-induced prolactin release. These results provide strong experimental evidence for the mechanism of “intracellular calcium mobilization mediates TNF-α to promote prolactin secretion.” (70).

## 6 A bidirectional neuroendocrine–metabolic network

Hyperprolactinemia is closely related to obesity at both epidemiological and pathogenic levels. We hypothesize that hyperprolactinemia can promote the occurrence and development of obesity through the synergistic dysregulation of multiple physiological systems. In the central nervous system, elevated prolactin can activate neurons in key hypothalamic nuclei, including the arcuate nucleus, and upregulate the expression of appetite-stimulating genes. This enhances appetite and promotes the reward-driven intake of high-fat diets. Simultaneously, prolactin can induce leptin resistance, further disrupting the central energy homeostasis. In peripheral tissues, prolactin promotes adipogenesis, enhances adipocyte differentiation, and increases lipid accumulation by activating key transcriptional regulators—including CCAAT/enhancer-binding protein β (C/EBPβ) and peroxisome proliferator-activated receptor γ (PPARγ). Furthermore, hyperprolactinemia is strongly associated with the pathological albinism of BAT, inhibition of adiponectin secretion, decreased insulin sensitivity, and glucose metabolism disorders. Meanwhile, prolactin-mediated HPG axis dysfunction can lead to sex hormone imbalance, resulting in unfavorable fat redistribution and a decreased basal metabolic rate. Finally, repeated observations of prolactin circadian rhythm disruption and weakened dopamine D2 receptor signaling in obese individuals further support the bidirectional neuroendocrine interaction between prolactin and metabolic regulation.

In contrast, obesity can also conversely promote elevated prolactin levels through several interconnected mechanisms, including the following: (1) ectopic prolactin synthesis and secretion in adipose tissue—especially visceral adipose tissue; (2) enhanced aromatase activity in adipose tissue, leading to increased local and systemic estrogen levels, thereby stimulating pituitary prolactinocytes to synthesize and secrete prolactin; and (3) obesity-related adipose tissue expansion triggers a chronic low-grade systemic inflammatory response, in which pro-inflammatory cytokines (such as IL-6 and TNF-α) can directly act on the anterior pituitary gland, enhancing prolactin secretion.

In summary, evidence from animal models, *in vitro* experimental systems, and clinical observational studies support our hypothesis of a possible correlation between hyperprolactinemia and obesity. At present, causal inference regarding prolactin-mediated metabolic effects in humans remains unproven, and most mechanistic insights derive from animal and experimental models. Furthermore, this association remains heterogeneous; not all obese individuals develop hyperprolactinemia, nor do all patients with hyperprolactinemia inevitably become obese. Current evidence is primarily based on animal experiments, and caution is required when extrapolating rodent data to human physiology. Although potential interaction mechanisms have been proposed, the interrelationships between these pathways and their relative contributions to different physiological or pathological backgrounds require further clarification. To determine the causal direction and assess the potential therapeutic value of regulating regulation in obesity-related metabolic diseases, further in-depth mechanistic and translational medicine research is needed to clarify the causal relationships and identify potential intervention targets within this endocrine-metabolic network.

Furthermore, although substantial evidence supports the association between pathological hyperprolactinemia and adverse metabolic outcomes, current research suggests that the relationship between prolactin and metabolic risk is not a simple linear model. The consensus generally defines prolactin deficiency as a serum prolactin level below 5 μg/L, a threshold primarily based on reproductive outcomes; however, recent clinical and observational studies have shown that prolactin levels below 7 μg/L may also be associated with unfavorable metabolic phenotypes. In contrast, prolactin levels within the physiological range, particularly the proposed HomeoFIT-PRL range (approximately 7–100 μg/L), are associated with more favorable metabolic indicators. However, this concept has not yet been incorporated into current clinical practice guidelines. At present, it is merely a theoretical framework for promoting further research and discussion, and should not be used as a basis for clinical decision-making. PRL levels within the physiological or “metabolic” range can promote β-cell proliferation and survival, improve insulin sensitivity, inhibit gluconeogenesis, optimize lipid distribution, and reduce the risk of fatty liver disease (20). In summary, this evidence supports a non-linear, approximately U-shaped relationship between prolactin and metabolic homeostasis: both prolactin deficiency (<7 μg/L) and significant hyperprolactinemia (>100 μg/L) may impair glucose and lipid metabolism, while moderate increases within the physiological range may have a metabolic protective effect (2).

## 7 Clinical implications and future directions

### 7.1 Therapeutic considerations in patients with obesity and hyperprolactinemia

Currently, weight management strategies are divided into three categories: lifestyle interventions, drug therapy, and metabolic/bariatric surgeries. Lifestyle interventions founded on evidence-based dietary adjustments and regular physical activity are safe, widely applicable, and are the cornerstone of obesity management. However, the weight-loss effect is usually limited, and long-term adherence remains a major challenge. Drug therapy (such as glucagon-like peptide-1 receptor agonists and GLP-1 RAs) can achieve significant weight loss and improve metabolic complications, including hyperglycemia; however, the application is limited by indications and usually needs to be combined with lifestyle interventions to reduce the risk of weight rebound. Bariatric surgery can result in significant and lasting weight loss in suitable populations; however, it is accompanied by higher surgical risks, potential long-term complications, and late weight rebound in some patients (71).

Correcting hyperprolactinemia may be an adjunct strategy for the treatment of obesity. Dopamine receptor agonists are the standard treatment for hyperprolactinemia, and can effectively reduce and restore abnormally high prolactin levels. Clinical observational studies and meta-analyses have shown that metabolic indicators, such as BMI and body fat percentage, can be improved in patients with prolactinomas treated with dopamine agonists (72). However, these drugs are not recommended as first-line treatment for obesity or abnormal glucose metabolism (73). These potential metabolic benefits have been primarily demonstrated in patients with diagnosed pathological hyperprolactinemia, and dopamine agonists are not recommended for the treatment of obesity in patients with normoprolactinemia. Current evidence supports the potential value of correcting hyperprolactinemia as an adjunct intervention for obesity in specific populations; however, its causal relationship and clinical translational significance still need to be verified by more mechanistic and interventional studies.

In addition, clinical observational studies suggest that weight-loss intervention is associated with a decrease in circulating prolactin levels in individuals with hyperprolactinemia. Obese women who received a low-calorie diet intervention and achieved significant weight loss experienced a significant reduction in pulsatile prolactin secretion. Obese women experience a decrease in the magnitude of prolactin bursts after significant weight loss, whereas the frequency of bursts remains unchanged. However, even after significant weight loss, prolactin levels are often higher than those in healthy controls. These results suggest that weight loss may improve obesity-related hyperprolactinemia to some extent, but does not always restore prolactin secretion to the normal physiological range (74). Existing evidence comes mainly from observational clinical studies. The magnitude of the effect, interindividual differences, and specific mechanisms of prolactin reduction have not yet been systematically elucidated. In particular, it is unclear which molecular and neuroendocrine pathways affect prolactin regulation. Therefore, weight-loss interventions should be considered an adjunct strategy for regulating prolactin rather than a direct means of treating hyperprolactinemia. However, further research is required to clarify these relationships and guide evidence-based clinical practice.

Circadian rhythm regulation may play an important role in the relationship between prolactin and metabolic health. Phase advancement, amplitude reduction, or decreased stability of the prolactin circadian rhythm (e.g., the prolactin peak occurring earlier than the cortisol peak) may promote fat accumulation and metabolic abnormalities (75). However, this hypothesis is based on experimental studies and cross-species comparative data, and direct causal evidence in humans remains limited. It is currently unclear what specific mechanisms prolactin rhythm disorders affect appetite regulation, insulin sensitivity, energy expenditure, or adipose tissue function. Therefore, whether interventions targeting the prolactin circadian rhythm (such as behavioral modifications, time-based therapy, or pharmacological interventions) can bring about metabolic benefits remains speculative. Future human studies combining time-series hormone monitoring are urgently needed to elucidate the role of prolactin rhythm dynamics in metabolic regulation and its potential clinical value.

Currently, there is no consensus supporting routine prolactin screening in all obese individuals. Existing evidence does not recommend prolactin testing as a universal examination for patients with obesity. Instead, prolactin testing should be based on clinical indications, focusing on signs of hyperprolactinemia or pituitary lesions. Specifically, prolactin levels should be considered in obese individuals with typical hyperprolactinemia, including galactorrhea, reproductive or gonadal endocrine dysfunction (such as amenorrhea, oligomenorrhea, infertility, decreased libido, or erectile dysfunction), and symptoms suggestive of sellar lesions (such as persistent headaches, visual field defects, or cranial nerve involvement). In addition, prolactin testing is also reasonable in certain specific clinical situations, such as (i) when standard weight loss and metabolic interventions fail to achieve the expected clinical or biochemical improvement; (ii) when high-risk factors are present, such as known or suspected pituitary disease, a history of traumatic brain injury or radiation therapy, or long-term use of dopamine receptor antagonists; and (iii) when unexplained systemic or endocrine abnormalities remain after comprehensive evaluation. Importantly, these recommendations are based primarily on clinical experience and observational research evidence. Therefore, prolactin testing in obese individuals should be conducted using an individualized approach, and the results should be carefully interpreted in conjunction with the overall endocrine and metabolic context.

### 7.2 Unanswered questions and research priorities

Several key questions remained unanswered. Obesity is highly heterogeneous, but it is unclear whether abnormal prolactin regulation is more likely to occur in specific obesity phenotypes, such as obesity characterized by visceral fat accumulation or insulin resistance, or obesity accompanied by a chronic low-grade inflammatory state. Identifying patient subgroups with the most significant prolactin changes may help optimize risk stratification and guide more precise endocrine assessments. Furthermore, although substantial evidence supports an association between pathological hyperprolactinemia and adverse metabolic outcomes, recent studies have suggested that both excessively high and low prolactin levels may have adverse effects on metabolism. Whether an optimal physiological prolactin range is conducive for maintaining energy balance and glucose homeostasis remains largely based on observational and experimental evidence, with limited research and definitive conclusions. Although the expression of extrapituitary prolactin in adipose tissue and immune cells has been reported, its quantitative contribution to circulating prolactin levels and functional significance in human metabolic regulation remain unclear. Clarifying whether locally produced prolactin primarily exerts its effects through paracrine or endocrine pathways is crucial for refining PRL-metabolic interaction models. Future research should integrate longitudinal population studies, mechanistic experiments, and circadian rhythm assessments to determine whether prolactin should be considered a driver, regulator, or biomarker of obesity-related metabolic disorders, thereby providing a theoretical basis for more refined and individualized clinical strategies.

## 8 Conclusions

We systematically summarize current research progress on the interaction mechanisms between hyperprolactinemia and obesity, focusing on the biological characteristics of prolactin, clinical phenotypes of hyperprolactinemia combined with obesity, and key molecules and physiological pathways involved in their interaction. Furthermore, we critically evaluated current clinical management strategies. We found that moderately elevated prolactin levels within the normal range were associated with an improved metabolic status, whereas both hyperprolactinemia and hypoprolactinemia were associated with adverse metabolic outcomes. Disruptions in the prolactin rhythm are also involved in the development and progression of metabolic disorders.

Despite the many insights provided by the existing research, many unknowns remain. Future research should focus on the prevention or treatment of metabolic diseases by regulating prolactin levels. The development of drugs that can regulate and reset prolactin rhythms would also be highly beneficial. Further clinical studies are needed to validate the causal relationship between prolactin and obesity, and to explore the specific mechanisms by which prolactin levels and rhythms are regulated to improve obesity and metabolic health. Future research will provide valuable insights into the use prolactin to improve patient outcomes and open new avenues for the treatment, intervention, and management of obesity and metabolic disorders.

## Glossary

Agrp: Agouti-related protein
AKT: Protein kinase B (AKT)
AMPK: AMP-activated protein kinase
ARC: Arcuate nucleus
BAT: Brown adipose tissue
BMI: Body mass index
cAMP: Cyclic adenosine monophosphate
C/EBPβ: CCAAT/enhancer-binding protein beta
DA: Dopamine
DLN: Dorsolateral nucleus
DMN: Dorsomedial nucleus
D2R: Dopamine D2 receptor
E2: 17β-estradiol
EGF: Epidermal growth factor
FSH: Follicle-stimulating hormone
GAS: γ-Interferon activation sequence
GLP-1: Glucagon-like peptide-1
GLP-1 RAs: Glucagon-like peptide-1 receptor agonists
GnRH: Gonadotropin-releasing hormone
HDL-C: High-density lipoprotein cholesterol
HPG: Hypothalamic–pituitary–gonadal (axis)
HPRL: Hyperprolactinemia
ICV: Intraventricular (intracerebroventricular)
IL-1β: Interleukin-1 beta
IL-6: Interleukin-6
JAK2: Janus kinase 2
kDa: Kilodalton
LDL-C: Low-density lipoprotein cholesterol
LH: Luteinizing hormone
MAPK: Mitogen-activated protein kinase
MASLD: Metabolic dysfunction–associated fatty liver disease
PY: Neuropeptide Y
PI3K: Phosphoinositide 3-kinase
PPARγ: Peroxisome proliferator-activated receptor gamma
PRL: Prolactin
PRLR: Prolactin receptor
Ras: Rat sarcoma (Ras) signaling family
SCN: Suprachiasmatic nucleus
STAT: Signal transducer and activator of transcription
TIDA: Tuberoinfundibular dopaminergic
TNF-α: Tumor necrosis factor alpha
TRH: Thyrotropin-releasing hormone.

## References

[1] Yang H Lin J Li H Liu Z Chen X Chen Q . Prolactin is associated with insulin resistance and beta-cell dysfunction in infertile women with polycystic ovary syndrome. Front Endocrinol. (2021) 12:571229. doi: 10.3389/fendo.2021.571229. 33716958 PMC7947819

[2] Macotela Y Triebel J Clapp C . Time for a new perspective on prolactin in metabolism. Trends Endocrinol Metab. (2020) 31:276–86. doi: 10.1016/j.tem.2020.01.004. 32044206

[3] Glezer A Garmes HM Kasuki L Martins M Elias PCL Nogueira VDSN . Hyperprolactinemia in women: diagnostic approach. Rev Bras Ginecol Obstet. (2024) 46:e-FPS04. doi: 10.61622/rbgo/2024FPS04. 38765533 PMC11078114

[4] Sung H Siegel RL Torre LA Pearson-Stuttard J Islami F Fedewa SA . Global patterns in excess body weight and the associated cancer burden. CA Cancer J Clin. (2019) 69:88–112. doi: 10.3322/caac.21499. 30548482

[5] Marcelin G Gautier EL Clément K . Adipose tissue fibrosis in obesity: etiology and challenges. Annu Rev Physiol. (2022) 84:135–55. doi: 10.1146/annurev-physiol-060721-092930. 34752708

[6] Engin A . Reappraisal of adipose tissue inflammation in obesity. Adv Exp Med Biol. (2024) 1460:297–327. doi: 10.1007/978-3-031-63657-8_10. 39287856

[7] Daghestani MH Daghestani MH Warsy A El-Ansary A Omair MA Omair MA . Adverse effects of selected markers on the metabolic and endocrine profiles of obese women with and without PCOS. Front Endocrinol (Lausanne). (2021) 12:665446. doi: 10.3389/fendo.2021.665446. 34122339 PMC8188979

[8] Ke X Wang L Zhao Y Duan L Deng K Yao Y . Serum prolactin levels were positively related to metabolic indexes and disorders in male obese patients. Endocrine. (2024) 84:1097–107. doi: 10.1007/s12020-024-03743-1. 38396200

[9] Pirchio R Graziadio C Colao A Pivonello R Auriemma RS . Metabolic effects of prolactin. Front Endocrinol. (2022) 13:1015520. doi: 10.3389/fendo.2022.1015520. 36237192 PMC9552666

[10] Borba V Carrera-Bastos P Zandman-Goddard G Lucia A Shoenfeld Y . Prolactin’s paradox: Friend, foe, or both in immune regulation? Autoimmun Rev. (2024) 23:103643. doi: 10.1016/j.autrev.2024.103643. 39306220

[11] Bernard V Young J Binart N . Prolactin - a pleiotropic factor in health and disease. Nat Rev Endocrinol. (2019) 15:356–65. doi: 10.1038/s41574-019-0194-6. 30899100

[12] Ben-Jonathan N Mershon JL Allen DL Steinmetz RW . Extrapituitary prolactin: distribution, regulation, functions, and clinical aspects. Endocr Rev. (1996) 17:639–69. doi: 10.1210/edrv-17-6-639. 8969972

[13] Nagy E Berczi I . Hypophysectomized rats depend on residual prolactin for survival. Endocrinology. (1991) 128:2776–84. doi: 10.1210/endo-128-6-2776. 2036962

[14] Featherstone K White MRH Davis JRE . The prolactin gene: a paradigm of tissue-specific gene regulation with complex temporal transcription dynamics. J Neuroendocrinol. (2012) 24:977–90. doi: 10.1111/j.1365-2826.2012.02310.x. 22420298 PMC3505372

[15] Grattan DR Kokay IC . Prolactin: a pleiotropic neuroendocrine hormone. J Neuroendocrinol. (2008) 20:752–63. doi: 10.1111/j.1365-2826.2008.01736.x. 18601698

[16] Melmed S Casanueva FF Hoffman AR Kleinberg DL Montori VM Schlechte JA . Diagnosis and treatment of hyperprolactinemia: an Endocrine Society clinical practice guideline. J Clin Endocrinol Metab. (2011) 96:273–88. doi: 10.1210/jc.2010-1692. 21296991

[17] Freeman ME Kanyicska B Lerant A Nagy G . Prolactin: structure, function, and regulation of secretion. Physiol Rev. (2000) 80:1523–631. doi: 10.1152/physrev.2000.80.4.1523. 11015620

[18] Mendoza RA Grandner MA Elali LS Fernandez F-X . Concerning the circadian rhythms of prolactin, its secretion timing, and regulation of the affiliative mind. Neurosci Biobehav Rev. (2025) 179:106403. doi: 10.1016/j.neubiorev.2025.106403. 41061945 PMC12557833

[19] Chasseloup F Bernard V Chanson P . Prolactin: structure, receptors, and functions. Rev Endocr Metab Disord. (2024) 25:953–66. doi: 10.1007/s11154-024-09915-8. 39476210

[20] Wu T Duan Y Jiang J Gu T Zhang P Bi Y . A century of prolactin: emerging perspectives as a metabolic regulator. Diabetes Metab Res Rev. (2024) 40:e3836. doi: 10.1002/dmrr.3836. 39096246

[21] Bugge K Papaleo E Haxholm GW Hopper JTS Robinson CV Olsen JG . A combined computational and structural model of the full-length human prolactin receptor. Nat Commun. (2016) 7:11578. doi: 10.1038/ncomms11578. 27174498 PMC4869255

[22] Bernard V Young J Chanson P Binart N . New insights in prolactin: pathological implications. Nat Rev Endocrinol. (2015) 11:265–75. doi: 10.1038/nrendo.2015.36. 25781857

[23] Methods In Medicine CAM . Retracted: PRL/PRLR can promote insulin resistance by activating the JAK2/STAT5 signaling pathway. Comput Math Methods Med. (2023) 2023:9897853. doi: 10.1155/2023/9897853. 38077883 PMC10700885

[24] Costa-Brito AR Gonçalves I Santos CRA . The brain as a source and a target of prolactin in mammals. Neural Regener Res. (2022) 17:1695–702. doi: 10.4103/1673-5374.332124. 35017416 PMC8820687

[25] Freemark M Fleenor D Driscoll P Binart N Kelly P . Body weight and fat deposition in prolactin receptor-deficient mice. Endocrinology. (2001) 142:532–7. doi: 10.1210/endo.142.2.7979. 11159821

[26] Naef L Woodside B . Prolactin/Leptin interactions in the control of food intake in rats. Endocrinology. (2007) 148:5977–83. doi: 10.1210/en.2007-0442. 17872372

[27] Grattan DR . 60 years of neuroendocrinology: The hypothalamo-prolactin axis. J Endocrinol. (2015) 226:T101–122. doi: 10.1530/JOE-15-0213. 26101377 PMC4515538

[28] Trott JF Schennink A Petrie WK Manjarin R VanKlompenberg MK Hovey RC . Triennial lactation symposium: Prolactin: The multifaceted potentiator of mammary growth and function. J Anim Sci. (2012) 90:1674–86. doi: 10.2527/jas.2011-4682. 22205663

[29] Ben-Jonathan N Hugo E . Prolactin (PRL) in adipose tissue: regulation and functions. Adv Exp Med Biol. (2015) 846:1–35. doi: 10.1007/978-3-319-12114-7_1. 25472532

[30] Ramos-Román MA . Prolactin and lactation as modifiers of diabetes risk in gestational diabetes. Horm Metab Res. (2011) 43:593–600. doi: 10.1055/s-0031-1284353. 21823053

[31] Banerjee RR Cyphert HA Walker EM Chakravarthy H Peiris H Gu X . Gestational diabetes mellitus from inactivation of prolactin receptor and MafB in islet β-cells. Diabetes. (2016) 65:2331–41. doi: 10.2337/db15-1527. 27217483 PMC4955982

[32] Kok P Roelfsema F Frölich M Meinders AE Pijl H . Prolactin release is enhanced in proportion to excess visceral fat in obese women. J Clin Endocrinol Metab. (2004) 89:4445–9. doi: 10.1210/jc.2003-032184. 15356045

[33] Guo H Yang B Kiryu S Wang Q Yu D Sun Z . Evaluation of the relations between reproduction-related pituitary and ovarian hormones and abdominal fat area-related variables determined with computed tomography in overweight or obese women who have undergone bariatric surgery: a cross-sectional study. Quant Imaging Med Surg. (2023) 13:7065–76. doi: 10.21037/qims-22-1283. 37869350 PMC10585523

[34] Al Sabie F Tariq Z Erickson D Donegan D . Association between prolactinoma and body mass index. Endocr Pract. (2021) 27:312–7. doi: 10.1016/j.eprac.2020.09.001. 33720014

[35] Galluzzi F Salti R Stagi S La Cauza F Chiarelli F . Reversible weight gain and prolactin levels--long-term follow-up in childhood. J Pediatr Endocrinol Metab. (2005) 18:921–4. doi: 10.1515/JPEM.2005.18.9.921. 16279371

[36] Nunes MC Sobrinho LG Calhaz-Jorge C Santos MA Mauricio JC Sousa MF . Psychosomatic factors in patients with hyperprolactinemia and/or galactorrhea. Obstet Gynecol. (1980) 55:591–5. 7189268

[37] Greenman Y Tordjman K Stern N . Increased body weight associated with prolactin secreting pituitary adenomas: weight loss with normalization of prolactin levels. Clin Endocrinol (Oxf). (1998) 48:547–53. doi: 10.1046/j.1365-2265.1998.00403.x. 9666865

[38] Colao A Sarno AD Cappabianca P Briganti F Pivonello R Somma CD . Gender differences in the prevalence, clinical features and response to cabergoline in hyperprolactinemia. Eur J Endocrinol. (2003) 148:325–31. doi: 10.1530/eje.0.1480325. 12611613

[39] Georgescu T Ladyman SR Brown RSE Grattan DR . Acute effects of prolactin on hypothalamic prolactin receptor expressing neurones in the mouse. J Neuroendocrinol. (2020) 32:e12908. doi: 10.1111/jne.12908. 33034148

[40] Sobrinho LG Horseman ND . Prolactin and human weight disturbances: a puzzling and neglected association. Rev Endocr Metab Disord. (2019) 20:197–206. doi: 10.1007/s11154-019-09503-1. 31062250

[41] Cornejo MP Lopez-Vicchi F de Winne C Pascual F Ornstein AM Reynaldo M . Chronic hyperprolactinemia is associated with enhanced high-fat diet binge eating in female mice. J Neuroendocrinol. (2026) 38:e70123. doi: 10.1111/jne.70123. 41404884

[42] Pala NA Laway BA Misgar RA Shah ZA Gojwari TA Dar TA . Profile of leptin, adiponectin, and body fat in patients with hyperprolactinemia: response to treatment with cabergoline. Indian J Endocrinol Metab. (2016) 20:177–81. doi: 10.4103/2230-8210.176346. 27042412 PMC4792017

[43] Gruzdeva O Borodkina D Uchasova E Dyleva Y Barbarash O . Leptin resistance: underlying mechanisms and diagnosis. Diabetes Metab Syndr Obes. (2019) 12:191–8. doi: 10.2147/DMSO.S182406. 30774404 PMC6354688

[44] Kirsch P Kunadia J Shah S Agrawal N . Metabolic effects of prolactin and the role of dopamine agonists: a review. Front Endocrinol (Lausanne). (2022) 13:1002320. doi: 10.3389/fendo.2022.1002320. 36246929 PMC9562454

[45] Nanbu-Wakao R Fujitani Y Masuho Y Muramatu M Wakao H . Prolactin enhances CCAAT enhancer-binding protein-beta (C/EBP beta) and peroxisome proliferator-activated receptor gamma (PPAR gamma) messenger RNA expression and stimulates adipogenic conversion of NIH-3T3 cells. Mol Endocrinol. (2000) 14:307–16. doi: 10.1210/mend.14.2.0420. 10674402

[46] Le JA Wilson HM Shehu A Devi YS Aguilar T Gibori G . Prolactin activation of the long form of its cognate receptor causes increased visceral fat and obesity in males as shown in transgenic mice expressing only this receptor subtype. Horm Metab Res. (2011) 43:931–7. doi: 10.1055/s-0031-1291182. 21989556 PMC3799815

[47] Corona G Rastrelli G Comeglio P Guaraldi F Mazzatenta D Sforza A . The metabolic role of prolactin: systematic review, meta-analysis and preclinical considerations. Expert Rev Endocrinol Metab. (2022) 17:533–45. doi: 10.1080/17446651.2022.2144829. 36447418

[48] Lopez-Vicchi F De Winne C Ornstein AM Sorianello E Toneatto J Becu-Villalobos D . Severe hyperprolactinemia promotes brown adipose tissue whitening and aggravates high fat diet induced metabolic imbalance. Front Endocrinol (Lausanne). (2022) 13:883092. doi: 10.3389/fendo.2022.883092. 35757410 PMC9226672

[49] Lee S-H Park S-Y Choi CS . Insulin resistance: from mechanisms to therapeutic strategies. Diabetes Metab J. (2022) 46:15–37. doi: 10.4093/dmj.2021.0280. 34965646 PMC8831809

[50] Berinder K Nyström T Höybye C Hall K Hulting A-L . Insulin sensitivity and lipid profile in prolactinoma patients before and after normalization of prolactin by dopamine agonist therapy. Pituitary. (2011) 14:199–207. doi: 10.1007/s11102-010-0277-9. 21128120

[51] Ben-Jonathan N Hugo ER Brandebourg TD LaPensee CR . Focus on prolactin as a metabolic hormone. Trends Endocrinol Metab. (2006) 17:110–6. doi: 10.1016/j.tem.2006.02.005. 16517173

[52] Auriemma RS De Alcubierre D Pirchio R Pivonello R Colao A . Glucose abnormalities associated to prolactin secreting pituitary adenomas. Front Endocrinol (Lausanne). (2019) 10:327. doi: 10.3389/fendo.2019.00327. 31191454 PMC6540784

[53] Daimon M Kamba A Murakami H Mizushiri S Osonoi S Yamaichi M . Association between serum prolactin levels and insulin resistance in non-diabetic men. PloS One. (2017) 12:e0175204. doi: 10.1371/journal.pone.0175204. 28384295 PMC5383244

[54] Andereggen L Frey J Andres RH Luedi MM Gralla J Schubert GA . Impact of primary medical or surgical therapy on prolactinoma patients’ BMI and metabolic profile over the long-term. J Clin Transl Endocrinol. (2021) 24:100258. doi: 10.1016/j.jcte.2021.100258. 34195008 PMC8237353

[55] Therkelsen KE Abraham TM Pedley A Massaro JM Sutherland P Hoffmann U . Association between prolactin and incidence of cardiovascular risk factors in the Framingham Heart Study. J Am Heart Assoc. (2016) 5:e002640. doi: 10.1161/JAHA.115.002640. 26908403 PMC4802489

[56] Barber TM Kyrou I Kaltsas G Grossman AB Randeva HS Weickert MO . Mechanisms of central hypogonadism. Int J Mol Sci. (2021) 22:8217. doi: 10.3390/ijms22158217. 34360982 PMC8348115

[57] Donato J Frazão R . Interactions between prolactin and kisspeptin to control reproduction. Arch Endocrinol Metab. (2016) 60:587–95. doi: 10.1590/2359-3997000000230. 27901187 PMC10522168

[58] Demura R Ono M Demura H Shizume K Oouchi H . Prolactin directly inhibits basal as well as gonadotropin-stimulated secretion of progesterone and 17 beta-estradiol in the human ovary. J Clin Endocrinol Metab. (1982) 54:1246–50. doi: 10.1210/jcem-54-6-1246. 7076799

[59] Mahboobifard F Pourgholami MH Jorjani M Dargahi L Amiri M Sadeghi S . Estrogen as a key regulator of energy homeostasis and metabolic health. BioMed Pharmacother. (2022) 156:113808. doi: 10.1016/j.biopha.2022.113808. 36252357

[60] Lizcano F Guzmán G . Estrogen deficiency and the origin of obesity during menopause. BioMed Res Int. (2014) 2014:757461. doi: 10.1155/2014/757461. 24734243 PMC3964739

[61] Carrageta DF Oliveira PF Alves MG Monteiro MP . Obesity and male hypogonadism: Tales of a vicious cycle. Obes Rev. (2019) 20:1148–58. doi: 10.1111/obr.12863. 31035310

[62] Dabbous Z Atkin SL . Hyperprolactinaemia in male infertility: Clinical case scenarios. Arab J Urol. (2018) 16:44–52. doi: 10.1016/j.aju.2017.10.002. 29713535 PMC5922222

[63] Wang F Xie N Wu Y Zhang Q Zhu Y Dai M . Association between circadian rhythm disruption and polycystic ovary syndrome. Fertil Steril. (2021) 115:771–81. doi: 10.1016/j.fertnstert.2020.08.1425. 33358334

[64] Zhang P-Z Shi Y-H Guo Y-X Li Y-Y Yin H-L Wu T-Y . Social jetlag elicits fatty liver via perturbed circulating prolactin rhythm-mediated circadian remodeling of hepatic lipid metabolism. Mil Med Res. (2025) 12:29. doi: 10.1186/s40779-025-00609-z. 40462123 PMC12131380

[65] Hugo ER Borcherding DC Gersin KS Loftus J Ben-Jonathan N . Prolactin release by adipose explants, primary adipocytes, and LS14 adipocytes. J Clin Endocrinol Metab. (2008) 93:4006–12. doi: 10.1210/jc.2008-1172. 18647802 PMC2579649

[66] Leeners B Geary N Tobler PN Asarian L . Ovarian hormones and obesity. Hum Reprod Update. (2017) 23:300–21. doi: 10.1093/humupd/dmw045. 28333235 PMC5850121

[67] Ronchetti SA Miler EA Duvilanski BH Cabilla JP . Cadmium mimics estrogen-driven cell proliferation and prolactin secretion from anterior pituitary cells. PloS One. (2013) 8:e81101. doi: 10.1371/journal.pone.0081101. 24236210 PMC3827476

[68] Wang S Cao S Arhatte M Li D Shi Y Kurz S . Adipocyte Piezo1 mediates obesogenic adipogenesis through the FGF1/FGFR1 signaling pathway in mice. Nat Commun. (2020) 11:2303. doi: 10.1038/s41467-020-16026-w. 32385276 PMC7211025

[69] Yamaguchi M Koike K Yoshimoto Y Ikegami H Miyake A Tanizawa O . Effect of TNF-alpha on prolactin secretion from rat anterior pituitary and dopamine release from the hypothalamus: Comparison with the effect of interleukin-1 beta. Endocrinol Jpn. (1991) 38:357–61. doi: 10.1507/endocrj1954.38.357. 1802676

[70] Koike K Masumoto N Kasahara K Yamaguchi M Tasaka K Hirota K . Tumor necrosis factor-alpha stimulates prolactin release from anterior pituitary cells: A possible involvement of intracellular calcium mobilization. Endocrinology. (1991) 128:2785–90. doi: 10.1210/endo-128-6-2785. 2036963

[71] Aronne LJ Hall KD M Jakicic J Leibel RL Lowe MR Rosenbaum M . Describing the weight-reduced state: Physiology, behavior, and interventions. Obes (Silver Spring). (2021) 29 Suppl 1:S9–S24. doi: 10.1002/oby.23086. 33759395 PMC9022199

[72] Lisco G De Tullio A Iovino M Disoteo O Guastamacchia E Giagulli VA . Dopamine in the regulation of glucose homeostasis, pathogenesis of type 2 diabetes, and chronic conditions of impaired dopamine activity/metabolism: Implication for pathophysiological and therapeutic purposes. Biomedicines. (2023) 11:2993. doi: 10.3390/biomedicines11112993. 38001993 PMC10669051

[73] Simonds SE Cowley MA . Speed-dieting: Dopamine agonists promote weight loss. Nat Metab. (2019) 1:851–2. doi: 10.1038/s42255-019-0114-z. 32694744

[74] Kok P Roelfsema F Langendonk JG de Wit CC Frölich M Burggraaf J . Increased circadian prolactin release is blunted after body weight loss in obese premenopausal women. Am J Physiol Endocrinol Metab. (2006) 290:E218–224. doi: 10.1152/ajpendo.00156.2005. 16144819

[75] Roelfsema F Pijl H . Phase difference between serum prolactin and cortisol rhythms is related to body mass index in humans. J Clin Endocrinol Metab. (2012) 97:E2293–2296. doi: 10.1210/jc.2012-2404. 23012388

